# Editorial: Sustaining the implementation of evidence-based interventions in clinical and community settings

**DOI:** 10.3389/frhs.2023.1176023

**Published:** 2023-03-24

**Authors:** Nicole Nathan, Rachel C. Shelton, Celia V. Laur, Maji Hailemariam, Alix Hall

**Affiliations:** ^1^ Hunter New England Population Health, Hunter New England Area Health Service, Newcastle, NSW, Australia; ^2^ School of Medicine and Public Health, The University of Newcastle, Newcastle, NSW, Australia; ^3^ National Centre of Implementation Science, Newcastle, NSW, Australia; ^4^ Hunter Medical Research Institute, Newcastle, NSW, Australia; ^5^ Department of Sociomedical Sciences, Mailman School of Public Health, Columbia University, New York, NY, United States; ^6^ Women’s College Hospital Institute for Health System Solutions and Virtual Care, Toronto, ON, Canada; ^7^ Institute of Health Policy, Management and Evaluation, University of Toronto, Toronto, ON, Canada; ^8^ Department of Obstetrics, Gynaecology and Reproductive Biology, College of Human Medicine, Michigan State University, East Lansing, MI, United States

**Keywords:** sustainability, sustainment, future direction, community settings, clinical settings, sustainability strategies, measurement

**Editorial on the Research Topic** Sustaining the implementation of evidence-based interventions in clinical and community settings

## Introduction

For the last few decades, the science of how best to implement effective Evidence Based Interventions (EBIs) has become an important focus in health services research (1). Accordingly, we have seen significant improvements in the adoption and implementation of EBIs in both clinical and community settings, leading to improvements in health outcomes and healthcare delivery (2, 3). For example, a review by Cassidy et al. of 41 studies found that multi-strategy interventions were effective at implementing guidelines in nursing which had positive effects on patient health status outcomes (3).

The public health impact of such EBIs and their equitable delivery and impact is however dependent upon how well and for how long they are implemented (4). For example, modelling of Australian government obesity targets suggests it will require the sustained implementation of a number of effective EBIs for at least a decade to achieve these targets (5). However, evidence from systematic reviews suggest that only 23% of public health and clinical interventions are sustained two years after initial implementation resulting in reduced health benefit (6). This has led sustainability to be identified as “one of the most significant translational research problems of our time” (7). As less than 1% of research in the last decade has focused on sustainability (6, 8), this Research Topic set out to encourage research that has explored the issue of sustainability (sustained impact and delivery of EBIs over time) across a diverse range of settings and health topics. Collectively, these 14 papers offer some key insights into the challenges, possible solutions, and future research needed within the field of sustainability science including:

1. *Design for sustainment from the outset.*

A common theme within this Research Topic was the importance of planning for sustainability from the start of implementation efforts. Two important pre-implementation steps identified were: (i) involving key knowledge users early in the planning process to ensure that they value and have a sense of ownership over the intervention; and (ii) integrating the EBI into existing systems and structures within the organisation.

For example, the study by Swindle et al. found in a survey of Early Care and Education Directors who had implemented a nutrition intervention that continued use of the program was associated with (i) their perception that the EBI was better than alternative programs and (ii) the EBI was integrated into centre schedules and routines. In our paper (Nathan et al.), published in this Research Topic, we propose a compilation of strategies that could be considered to support sustainability, highlighting that many should be considered at earlier phases of the implementation process, rather than planning for and instigating sustainability during the sustainability phase only. Despite the importance of planning early for sustainability it does not yet seem to be common practice, as shown in the review by Engelhart et al. where planning for sustainability was often not addressed or was deficient. If we are to successfully sustain EBI delivery post-active implementation, we need to work towards actively planning for sustainability as early as possible.

2. *Engaged and supportive leadership team is essential for sustainability*

Several studies included in this Research Topic identified the vital role leadership support plays in the sustainability of EBIs, including those by Nadalin Penno et al., Agulnik et al. Love et al. and McLoughlin et al. Demonstrable support from organisational leadership seems particularly pertinent for successful sustainability of EBIs. Ways leadership can actively engage and demonstrate their support for EBI sustainment were identified in the study by Agulnik et al. and included strategies such as: provision of physical resources (e.g., financial support, equipment), active support for implementation (e.g., ensure staff are trained in the EBI or ratify institutional policies for the EBI) or publicly endorsing the EBI (e.g., acknowledging staff for their work or attending team meetings). Future efforts to sustain EBIs should endeavour to actively engage organisational leaders early, and may consider encouraging leadership to employ such strategies to demonstrate their support for EBI sustainment.

3. *Ongoing access to education and training is needed to sustain EBI implementation*

Training is a common implementation strategy used to enhance the knowledge and skills of clinicians, or those working in community settings, to effectively implement EBIs. Systematic reviews of the sustainability of EBIs have however consistently found that staff turnover is a key determinant to EBI sustainment (9, 10). High staff turnover can significantly impact an organisations ability to continue to deliver an EBI, as there is a loss of corporate knowledge when trained staff leave, while new staff may have limited understanding of the need for the EBI or competence to effectively deliver it. Implementation researchers and practitioners may therefore need to consider how, after once active implementation support has ceased, new staff to the organisation will be trained to deliver the intervention with enough fidelity to ensure sustained delivery and impact. The importance of staff training to sustainability was identified by multiple studies in this Research Topic, including those conducted by: Rakhra et al. Siegal et al. Love et al. and McLoughlin et al. Strategies that may be effective include incorporating training into orientation processes for new staff and offering booster sessions for existing staff, employing low cost training modalities i.e., online training, creating handover manuals or forming communities of practice within and between organisations (10, 11).

4. *Routine monitoring of EBI delivery can facilitate sustainability*

Monitoring the continued and equitable delivery of an EBI once the implementation phase has ended is crucial for sustainability (12), although not yet common practice. This highlights a current limitation of the field to routinely monitor and report EBI delivery to facilitate sustainability. Whilst we know that most EBI implementation attenuates over time (13), little is known as to when or how quickly such reductions occur. Routine monitoring of EBI implementation would enable agencies to identify when and what kind of support may be needed in order to ensure that the EBI continues to be delivered over time, and reaches a diverse range of settings and populations. Central to any monitoring system is the use of valid and reliable measures (13).

However, inconsistencies in how sustainability is defined and measured seems to be a significant limitation in the field. For example, the review by Engelhart et al. found that the variation in the definitions and methods used to measure sustainability impacted the ability to gather high quality and generalizable information on the sustainability of breastfeeding interventions in low- and middle-income countries. To ensure accurate monitoring and understanding of sustainability, it is imperative the field moves towards developing and using valid, reliable and standardised measures of sustainability (14). A recent comprehensive review by Hall et al. evaluated various measures of sustainability and sustainability determinants relevant to clinical and community settings (13). Efforts like these may help guide those developing monitoring systems in their selection of robust, pragmatic measures.

## Conclusion

Failure to sustain implementation of effective EBIs wastes the considerable health system investment required to achieve initial implementation, often results in organisations regressing to pre-implementation levels, and reduces partners trust and willingness to engage in future initiatives (1). Therefore, as policy-makers, practitioners and researchers we have a responsibility to ensure that we think carefully about the EBIs we select for implementation and then how (if at all) we plan to support its ongoing implementation before we invest scarce public health dollars into its implementation. Encouragingly, this Research Topic suggests that work is being done across contexts and health issues to answer pressing issues in the field. Collectively, the included studies highlights some important future directions that those working in the field may consider for research and practice, in particular: identify, describe and rigorously test the effectiveness of sustainability strategies in clinical and community settings, determine methods or processes for establishing monitoring systems and describe how existing or new measures or tools are applied in sustainability research.
